# Looking for related posts on GitHub discussions

**DOI:** 10.7717/peerj-cs.1567

**Published:** 2023-11-09

**Authors:** Marcia Lima, Igor Steinmacher, Denae Ford, Evangeline Liu, Grace Vorreuter, Tayana Conte, Bruno Gadelha

**Affiliations:** 1 Department of Computer Science, Amazonas State University (UEA), Manaus, Amazonas, Brazil; 2 Institute of Computing (IComp), Federal University of Amazonas (UFAM), Manaus, Amazonas, Brazil; 3 School of Informatics, Computing, and Cyber Systems, Northern Arizona University (NAU), Flagstaff, Arizona, USA; 4 Department of Microsoft Research Lab—Redmond, Microsoft Research, Redmond, WA, USA; 5 GitHub Discussions Department, GitHub, Upstate NY, NY, USA

**Keywords:** Sentence-BERT, Software teams interaction, Communication tool, Knowledge sharing, GitHub Discussions, Related posts

## Abstract

Software teams increasingly adopt different tools and communication channels to aid the software collaborative development model and coordinate tasks. Among such resources, software development forums have become widely used by developers. Such environments enable developers to get and share technical information quickly. In line with this trend, GitHub announced GitHub Discussions—a native forum to facilitate collaborative discussions between users and members of communities hosted on the platform. Since GitHub Discussions is a software development forum, it faces challenges similar to those faced by systems used for asynchronous communication, including the problems caused by related posts (duplicated and near-duplicated posts). These related posts can add noise to the platform and compromise project knowledge sharing. Hence, this article addresses the problem of detecting related posts on GitHub Discussions. To achieve this, we propose an approach based on a Sentence-BERT pre-trained general-purpose model: the *RD-Detector*. We evaluated *RD-Detector* using data from three communities hosted in GitHub. Our dataset comprises 16,048 discussion posts. Three maintainers and three Software Engineering (SE) researchers manually evaluated the *RD-Detector* results, achieving 77–100% of precision and 66% of recall. In addition, maintainers pointed out practical applications of the approach, such as providing knowledge to support merging the discussion posts and converting the posts to comments on other related posts. Maintainers can benefit from *RD-Detector* to address the labor-intensive task of manually detecting related posts.

## Introduction

Software engineering teams actively adopt social communication means to support collaborative software development and coordinate team members’ tasks (Storey et al., 2014, 2016; Tantisuwankul et al., 2019). Teams use such resources to communicate, learn, answer questions, obtain and give feedback, show results, manage, and coordinate activities (Storey et al., 2016). E-mail, chats, and forums are examples of collaborative social media that support software teams’ communications (Storey et al., 2014, 2016; Pérez-Soler, Guerra & de Lara, 2018). In recent years, software development Question & Answering (Q&A) forums have increasingly attracted different users’ attention and have become widely used by software developers (Ford et al., 2018; Wang, Zhang & Jiang, 2020; Pei et al., 2021). Developers rely on such platforms to quickly find answers to technical questions (Mamykina et al., 2011; Zhang et al., 2017), impacting the software development process (Yazdaninia, Lo & Sami, 2021).

In accordance with this context, GitHub announced GitHub Discussions in 2020. To be “A new way for software communities to collaborate outside the codebase” (Niyogi, 2020), Discussions provides opportunities for communities to interact and discuss project-specific issues collaboratively. GitHub Discussions is a place where communities can talk about work, ask questions, plan new releases, request code reviews, make announcements, disclose information, recruit contributors, get insights into the project, feature important information, or simply chat (Hata et al., 2022). However, Discussions should be concerned about the quality. A decaying quality is a trouble for any Q&A forum. Silva, Paixão & de Almeida Maia (2018) point out that repeated questions put at risk that quality.

Since data duplication is a widely recognized issue, previous studies have tackled the problem of deduplication in communication channels used by software developers. These channels include bug-tracking and issue-tracking systems, discussion forums, and collaborative development platforms like GitHub (Zhang et al., 2018; Lima & Soares, 2022; Pei et al., 2021). Research highlights that duplicates can (1) pollute the forums with already-answered questions (Silva, Paixão & de Almeida Maia, 2018); (2) consume the time of experts, as they must manually analyze and look for duplicates (Zhang et al., 2017; Wang, Zhang & Jiang, 2020); and (3) make users wait unnecessarily for answers to questions that had been already asked and answered (Ahasanuzzaman et al., 2016). Since duplicates and near-duplicates are also well-known problems among GitHub Issues and Pull-requests (PRs) (Li et al., 2018; Wang et al., 2019; Lima & Soares, 2022), researchers also apply different efforts to detect duplicates on the GitHub platform. Duplicate posts on GitHub increase the maintenance costs (Yu et al., 2018), time spent on addressing redundant data (Lima & Soares, 2022), and can even frustrate developers who want to contribute continuously (Wang et al., 2019).

GitHub Discussions differs from other software development forums, such as Stack Overflow (https://stackoverflow.com/), because it integrates with the GitHub platform. Posts on GitHub Discussions cover a software project ecosystem (Hata et al., 2022); they aim to answer developers’ technical questions regardless of a specific software project (on GitHub, each project has its own forum). However, textual evidence collected from discussion threads (discussion first posts and comments) shows that the forum contains duplicate and near-duplicate posts (https://github.com/homebrew/discussions/discussions/1531, https://github.com/homebrew/discussions/discussions/707, https://github.com/vercel/next.js/discussions/22211). We conjecture GitHub Discussions users create duplicates or near-duplicates to (1) emphasize their need for help, (2) add new information to better detail the post’s main topic, and (3) ask for different solutions to the same issue. To deduplicate discussion posts, maintainers manually look for duplicates on the GitHub Discussions. Based on their previous knowledge, maintainers identify duplicates as new discussions arise. However, manual strategies are less effective than automatic ones and are prone to human subjectivity and imprecision (Ahasanuzzaman et al., 2016; Zhang et al., 2017; Wang, Zhang & Jiang, 2020).

Different approaches have already been proposed and evaluated to solve the problem of automatically identifying duplicate posts in developers’ communication channels. Such methods often use pre-labeled datasets to train or optimize the duplicate detection process (Kukkar et al., 2020; Zhang et al., 2020; Pei et al., 2021). However, the posts on GitHub Discussions are project-specific and not previously categorized using pre-defined topics. In addition, different project contexts emerge every time a community enables the Discussions forum feature on its repository (the GitHub platform hosts more than 200 million repositories (https://github.com)). The diversity of projects hosted on the platform and the project-context-based posts create opportunities and challenges to develop an approach to detect duplicate and near-duplicate discussion posts on GitHub Discussions.

Therefore, inspired by the context mentioned earlier, this research introduces the RD-Detector, an automated approach to detect related posts on GitHub Discussions. Specifically, this study considers related posts, those posts that are duplicates or near-duplicates, with detailed definitions provided in subsequent sections of this article.

More specifically, our research question (RQ) is:

**RQ:** Are general-purpose deep machine learning models applied to Natural Language Processing (NLP) problems effective in detecting related posts in the GitHub Discussions?

To do so, we propose the RD-Detector approach. RD-Detector uses a Sentence-BERT (SBERT) pre-trained general-purpose model (Reimers & Gurevych, 2019) to create sentence embedding representations of the discussions’ first posts (“discussion posts” or, simply, “posts”). Once the approach computes the sentence embeddings, it measures the semantic similarity of two posts using the cosine similarity. The RD-Detector also calculates a dynamic threshold value to determine whether the posts are related (including duplicates and near-duplicates). The approach differs from the previous ones as (1) it does not rely on pre-labeled datasets; (2) it does not rely on context-specific models; and (3) it can assess different software contexts hosted on GitHub. Those features are relevant due to the variety and particularities of project contexts and the project-specific posts. We evaluated RD-Detector on three real GitHub Discussions forums. The evaluation involved three OSS maintainers, one per project, and three Software Engineering (SE) researchers. They assessed the RD-Detector results by classifying pairs of related post candidates as either related or not. Additionally, they pointed out practical applications for detecting related posts in Discussions.

Our results show that we can use a general-purpose machine learning model for detecting related posts in GitHub communities. We evaluated the RD-Detector results in three different datasets, achieving 77% to 100% in terms of precision and 66% of recall. We highlight the imprecision of the term “related posts” as a limitation during the evaluation. To mitigate this limitation, we invited three OSS maintainers to judge the relatedness between candidates of related posts that required prior knowledge about the project. The general-purpose machine learning model also brought flexibility to the approach. As an advantage, we can point out that RD-Detector does not restrict to a specific software project context hosted on GitHub. The RD-Detector is a parameterizable approach. This design decision allows maintainers to set the RD-Detector parameters to navigate between results with higher or lower precision and recall according to their needs. Maintainers can benefit from the approach to minimize the work overhead in manually detecting related posts and the rework in answering the same question multiple times. Besides, the results support maintainers in tackling the platform pollution and the project knowledge-sharing degradation, as it occurs in different discussion threads. To our knowledge, this work is the first attempt to detect related posts in Discussions. The main contributions of this research include the following:

- The RD-Detector, an approach based on deep machine learning models to detect related posts on GitHub Discussions.
- Empirical evidence on using a general-purpose machine learning model to detect related posts created by software communities.
- Empirical evidence regarding the RD-Detector practical applications in communities from three OSS maintainers’ perspectives.

Since Discussions is a new feature within the GitHub platform, it brings opportunities to research how communities communicate. We reported our findings to the GitHub Discussions engineering team. They actively collaborated in discussing the research problem, contacting maintainers, and participating in monthly virtual meetings to discuss and validate our findings. Based on our results, the engineering team is testing specific changes to the Discussions interface. We envision our results supporting the GitHub team’s decision-making process to improve the forum features and promote its adoption.

## Background

OSS projects are highly distributed environments usually composed of self-directed development teams (Chen et al., 2013; Tantisuwankul et al., 2019). Therefore, knowledge sharing is a critical factor for the success of OSS teams (Chen et al., 2013; Tantisuwankul et al., 2019). Chen et al. (2013) point out that “knowledge sharing is an interactive cuing process in which knowledge provided by one team member becomes the cue for other members to retrieve relevant but different knowledge stored in their own memory.” Given that GitHub Discussions is a collaborative communication channel where communities ask questions, debate, and announce project-specific issues, we conjecture that Discussions is an online environment that promotes knowledge sharing in GitHub communities. Tantisuwankul et al. (2019) point out that the key to software projects’ success hosted on collaborative platforms such as GitHub is the communities’ interaction and project knowledge sharing.

This section presents the main concepts used in this research and previous works on detecting duplicates in developers’ communication channels. The “Communication within the GitHub” section describes GitHub Discussions (our object of study). The “Related work” section presents how researchers tackle duplicates or related posts in bug and issue-tracking systems, Q&A forums, and GitHub features.

### Communication within the GitHub

Open source software development teams use electronic means, such as emails (mailing lists), instant messaging, or forums, to conduct open and public discussions (Guzzi et al., 2013; Vasilescu et al., 2014; Storey et al., 2016). Those channels are considered a rich source of information about OSS development (Guzzi et al., 2013; Storey et al., 2016; Brisson, Noei & Lyons, 2020). Historically, mailing lists were the preferred channel for project communication within GitHub (Rigby & Hassan, 2007; Guzzi et al., 2013; Vasilescu et al., 2014). However, with the rise of other social communication means, researchers observed that this preference was changing (Guzzi et al., 2013; Vasilescu et al., 2014). Issues repositories and PRs threads play relevant roles in GitHub communities’ communication (Brisson, Noei & Lyons, 2020). However, to meet developers’ requests for an effective communication channel at their disposal for open-ended tasks such as brainstorming (Hata et al., 2022), the GitHub engineering team released Discussions. According to Hata et al. (2022) “the forum is one of the first attempts at addressing this request on the GitHub platform.”

GitHub Discussions is a feature of any public or private repository on GitHub (2022a). It facilitates collaborative discussions among maintainers and the community for a project on GitHub (2022a). GitHub company suggests using Discussions to ask and answer questions, share information, make announcements, and lead or participate in project-specific conversations (GitHub, 2022a). Discussions is a collaborative communication forum for maintainers, code and non-code contributors, newcomers, and users to discuss projects’ use, development, and updates in a single place without third-party tools (GitHub, 2022b, 2022a). Additionally, Discussions is the place where daily conversations can take place separated from discussions specifically targeted toward engineering teams (Issues and PRs) (Hata et al., 2022).

Users, maintainers, contributors, and newcomers can join in a conversation by creating, commenting, reacting, or reading a discussion post (GitHub, 2022a). Discussions users can also search for specific topics in discussion posts (GitHub, 2021b). To do so, they must specify keywords in the GitHub search engine. Users can restrict the search results to the discussion title, body text, or comments by applying correct qualifiers.

**Figure 1:**
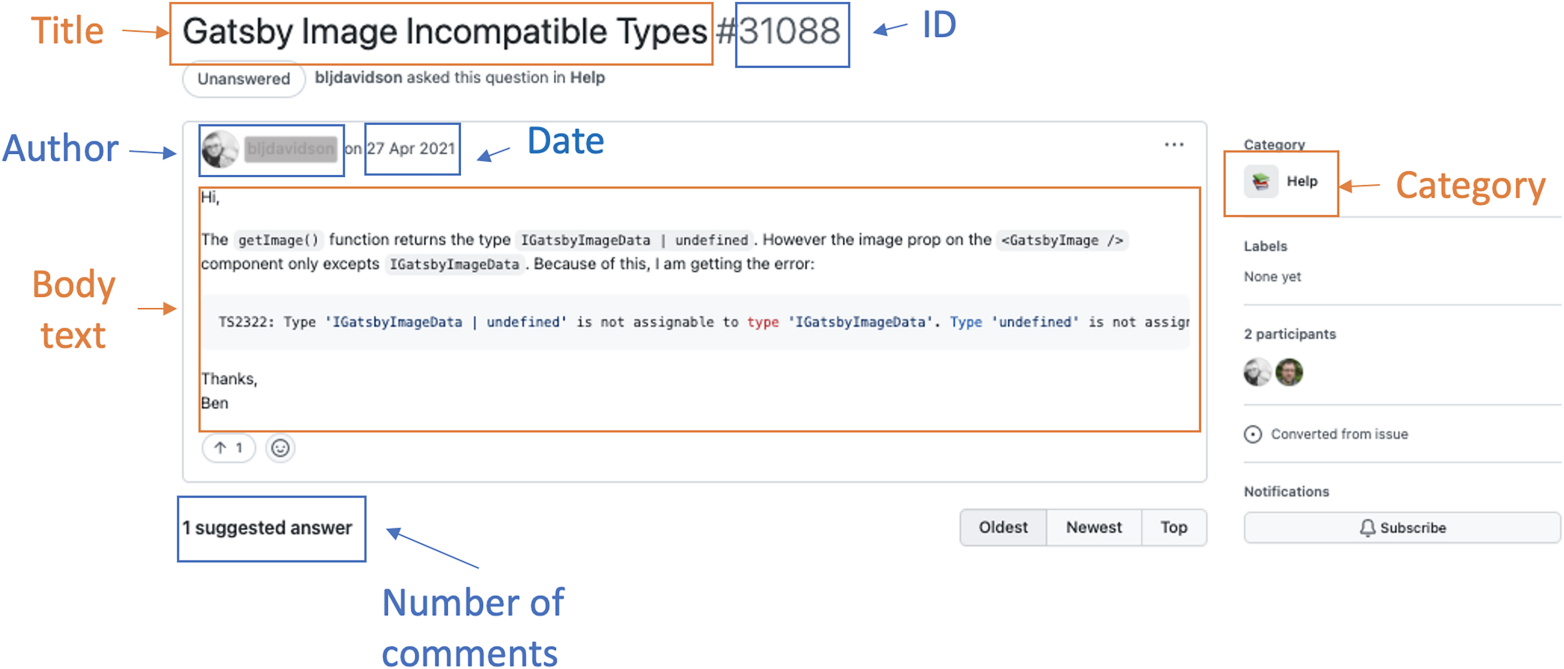
An example of a discussion post.

Authors must specify the discussion title, body text, and category to create new posts (Fig. 1). The category is a mandatory attribute. It helps organize conversations into predefined classes, allowing community members to chat in the right place and find posts with similar characteristics (GitHub, 2021a). Authorized members can define, create, or delete categories in a repository according to the project needs. By default, Discussions provides five types of categories: Announcements, Q&A, Ideas, Show and tell, and General (GitHub, 2021a). Maintainers can create Announcements posts to share project updates and news. Users can create Q&A posts to ask questions, suggest answers, and vote on the most appropriate feedback. Ideas posts can report or share ideas regarding the project improvements. Show and tell posts discuss relevant creations, experiments, or tests. Finally, General posts address any issue relevant to the project (GitHub, 2021a).

Maintainers report a positive acceptance of GitHub Discussions forum in the communities (https://github.blog/2021-08-17-github-discussions-out-of-beta/). They highlight that the forum enables the growth of the communities in the same place they use to collaborate, improve and increase the community members’ engagement, and separate the issues trackers from questions, feature requests, and general chatting. In addition, maintainers point out they can use the discussion threads to access historical data. They can keep track of the questions already asked, their proposed solutions, and the suggestions made. With this threading support, maintainers can individually address demands without losing them in broader discussions.

### Related work

Our research contributes to the growing literature on how software developers use communication channels. While Discussions is not the only primary means of communication for GitHub communities, our focus is aiding maintainers to identify related posts (including duplicate and near-duplicate posts) in the Discussions forum. By doing so, we aim to provide maintainers with the knowledge to carry out their tasks more efficiently.

Several studies have approached the deduplication problem in developers’ communication channels, such as bug-tracking and issue-tracking systems (Alipour, Hindle & Stroulia, 2013; Kukkar et al., 2020; Cooper et al., 2021), Q&A forums (Silva, Paixão & de Almeida Maia, 2018, Wang, Zhang & Jiang, 2020; Pei et al., 2021), and collaborative development platforms like GitHub (Yu et al., 2018; Wang et al., 2019; Lima & Soares, 2022;). Researchers use information retrieval (IR) techniques, machine learning (ML) algorithms, deep learning models (DLM), and even a combination of those to deduplicate data in software engineering contexts. Generally, researchers model the duplicate detection task as a ranking problem, a binary classification problem, or a combination of both. Ranking problems combine features to sort the top-k most similar documents (posts, texts, questions) (Liu, 2011) and predict duplicates. Classification problems classify pairs of documents into predefined categories (*e.g*., duplicates or non-duplicates) (Zhang et al., 2018).

Previous work highlights that detecting duplicate bug reports is crucial for identifying redundant issue reports in bug and issue-tracking systems (Alipour, Hindle & Stroulia, 2013; Cooper et al., 2021; Kukkar et al., 2020). Bug report deduplication is essential to avoid unnecessary work, which can incur significant human effort and time. Researchers claim thousands of duplicate bug reports are reported daily in typical bug and issue-tracking systems. In this sense, Runeson, Alexandersson & Nyholm (2007) investigated NLP techniques to identify duplicated Defect Reports. The authors claim that about 2/3 of the duplicates can be found using the NLP techniques. Alipour, Hindle & Stroulia (2013) investigated how contextual information can improve bug deduplication. The authors proposed a contextual bug-deduplication method evaluated on the Android ecosystem bug repository. The authors applied machine learning algorithms such as C4.5, K-NN (K Nearest Neighbours), Logistic Regression, and Naive Bayes. Their best result, including textual, categorical, and Android architectural context data, achieved an accuracy of 91% and 0.7284 of Kappa measure. Kukkar et al. (2020) proposed a CNN (convolutional neural network) based deep learning strategy for duplicate Bug report detection and classification. The proposed approach achieved an accuracy rate of 85% to 99% and a recall@k rate (the proportion of items successfully retrieved among the top k results) of 79–94%, setting $k = 20$. The authors used six publicly available datasets for the approach training and evaluation. Cooper et al. (2021) proposed TANGO, a method to address the challenge of detecting duplicate bug reports in screenshots and screen recordings. TANGO operates computer vision techniques, optical character recognition, and text retrieval to achieve its goal. The results showed that TANGO accurately ranked duplicate videos in the top-2 returned results in 83% of the tasks. The authors claim that TANGO saves almost 65.1% of humans’ time finding duplicates.

Regarding duplicates on developers’ Q&A forums, several studies also aim to detect duplicate questions in such forums (Zhang et al., 2015, 2018; Wang, Zhang & Jiang, 2020; Mohomed Jabbar et al., 2021; Pei et al., 2021). Zhang et al. (2015) proposed DupPredictor, an approach to detect potential duplicate questions in Stack Overflow. The DupPredictor combines the similarity scores of different features (titles, descriptions, latent topics, and tags). The authors evaluated the approach using a pre-labeled Stack Overflow dataset, which achieved a 63.8% recall rate. Ahasanuzzaman et al. (2016) and Zhang et al. (2017) addressed duplicate detection as a supervised classification problem, utilizing ML algorithms such as decision tree, KNN, SVM (support vector machines), logistic regression, random forest, and naive Bayes. The authors used a pre-labeled dataset to train and validate a classifier to detect duplicates in Stack Overflow and evaluated the proposed approach using the recall rate. Mizobuchi & Takayama (2017) created a Word2vec model using sentences extracted from Stack Overflow. The authors contrasted the model efficiency against a pre-labeled dataset they created. The best recall@k rate achieved was 43.13%, setting $k = 20$. Zhang et al. (2018) modeled duplicate detection as a “ranking-classification” problem over question pairs. The authors used a data dump of Stack Overflow to train and evaluate the proposed approach, achieving a precision of 75% to 86% and recall of 66% to 86%. Wang, Zhang & Jiang (2020) used deep learning techniques to detect duplicate questions in Stack Overflow. The authors proposed three different approaches based on Convolutional Neural Networks (CNN), Recurrent Neural Networks (RNN), and Long Short-Term Memory (LSTM) to identify duplicates. The authors also used a pre-labeled Stack Overflow dataset to train, evaluate, and test the classifiers and evaluated the approaches using the recall rate. The approach achieved 76–79% of recall@5. Mohomed Jabbar et al. (2021) proposed DeepDup, a deep learning model for duplicate question detection. The authors also use transfer learning techniques to improve the text-pair duplicates classification task, using pre-labeled datasets from the Stack Exchange sub-communities for Ubuntu and English to train and evaluate the approach, achieving an accuracy of almost 75–78%. Pei et al. (2021) proposed an Attention-based Sentence pair Interaction Model (ASIM) to predict the relationship between Stack Overflow questions. The authors used a Stack Overflow dump to train the software engineering specific domain. The proposed model achieved 82.10% precision and 82.28% recall rates. Finally, Gao, Wu & Xu (2022) proposed a method to detect duplicates in Stack Overflow that learns the semantics of a question by combining text features and source code features. The authors used word embedding and convolutional neural networks to extract textual features from questions and structural and semantic features from source code, achieving a recall rate of 68–79%.

Considering GitHub activities, previous work also highlighted the problem of duplicates in issue reports and PRs on GitHub (Li et al., 2018; Wang et al., 2019; Li et al., 2020). Li et al. (2018) analyzed explicit links in both issues reports and PRs. They reported the importance of such links in identifying duplicates. The authors analyzed 70,686 links that represented duplication relationships, from which 59.03% identified duplicate issues and 40.97% identified duplicate PRs. Wang et al. (2019) claim that the uncoordinated and distributed nature of creating issues and PRs leads to extensive data duplication. The authors claim that duplication considerably increases the workload of project reviewers and maintainers. To automatically identify duplicate PRs, the authors hypothesized that two PRs are more likely to be duplicated if they were created around the same time. They experimented on 26 open source repositories from GitHub with over 100,000 pairs of PRs. Their results showed that their assumption improved the baseline performance by 14.36% and 11.93% in terms of F1-score@1 and F1-score@5, respectively. Li et al. (2020) presented empirical evidence on the impact of duplicate PRs on the software development effort. They observed that the “inappropriateness of OSS contributors” work patterns and the drawbacks of their collaboration environment would result in duplicates. In addition, researchers have been proposing different approaches to address duplicates in GitHub (Li et al., 2017; Yu et al., 2018; Ren et al., 2019; Zhang et al., 2020). Li et al. (2017) and Ren et al. (2019) proposed automatic approaches based on traditional Information Retrieval (IR) and NLP techniques to detect duplicates in PRs. The approach proposed by Li et al. (2017) achieved a recall@20 of 55.3–71.0%. The approach proposed by Ren et al. (2019) achieved the best precision rate of 64.3%. Yu et al. (2018) constructed a dataset of historical duplicate PRs extracted from projects on GitHub-the DupPR dataset-by using a semi-automatic approach. The recall@k rate achieved was nearly 70% when the k was set to 20. Zhang et al. (2020) proposed the iLinker, an approach to detect related issues in GitHub. The authors trained iLinker to learn the embedding *corpus* and models from the project issue text. The approach achieved recall@5 of 38–51% and recall@10 of 45–61%. Finally, Li et al. (2021) present an approach that combines the *TF*−*IDF* and deep learning techniques to detect duplicate contributions in the pull-based model at the time of submission. The method produces a list of candidate duplicate contributions most similar to the new contribution, given the combined textual and similarity scores. Evaluation results show that 83.4% of the duplicates can be identified when using the combined textual and similarity scores, 54.8% when using only textual similarity, and 78.2% when using only similarity scores. Table 1 shows an overview of the pertinent literature.

**Table 1:** Summarization of the related works.

Research focus	Reference	How did the authors model the problem?	Dataset	*Precision (P)*	Recall (R)	*Accuracy*	Comments
Bug reports	Runeson, Alexandersson & Nyholm (2007)	Ranking problem	Defect reports from development projects.		24–42%		The approach uses NLP techniques.
	Alipour, Hindle & Stroulia (2013)	Classification task	Android ecosystem bug repository.			91%	The approach uses ML algorithms.
	Kukkar et al. (2020)	Classification task	Six datasets from (Lazar, Ritchey & Sharif, 2014; Lerch & Mezini, 2013)		79–94% R@20	85–99%	The approach is a CNN-based strategy.
	Cooper et al. (2021)	Ranking problem	RICO dataset (Deka et al., 2017)			83% top-2	The approach uses computer vision, optical recognition, and text retrieval techniques.
Q&A forums	Zhang et al. (2015)	Ranking problem	Pre-labeled Stack Overflow database		64% R@20		The approach combines the similarity scores of four features
	Ahasanuzzaman et al. (2016)	Classification task	Pre-labeled Stack Overflow database		66% R@20		The approach is a supervised classification strategy.
	Zhang et al. (2017)	Classification task	Pre-labeled Stack Overflow database		87%		The approach is based on ML algorithms.
	Mizobuchi & Takayama (2017)	Ranking problem	Pre-labeled Stack Overflow database		43% R@20		The approach is based on Word2vec models.
	Zhang et al. (2018)	Ranking-classification task	Pre-labeled Stack Overflow database	75–86%	66–86%		The approach uses rank strategies, deep learning, and IR techniques.
	Wang, Zhang & Jiang (2020)	Classification task	Pre-labeled Stack Overflow database		76–79% R@5		The approach is based on CNNs, RNNs, and LSTMs
	Mohomed Jabbar et al. (2021)	Classification task	Pre-labeled datasets from the Stack Exchange sub-communities			75–78%	The approach is based on deep learning and transfer learning techniques
	Pei et al. (2021)	Classification task	Pre-labeled Stack Overflow database	82%	82%		The approach is based on an Attention-based Sentence and ASIM model
	Gao, Wu & Xu, 2022	Classification task	Pre-labeled Stack Overflow database		68–79%		The approach is based on word embedding and CNNs
GitHub activities	Wang et al. (2019)	Classification task	DupPR (Yu et al., 2018)	73% P@1	65% R@1		The approach is based on AdaBoost algorithm.
	Li et al. (2017)	Ranking problem	The authors constructed a dataset of duplicate PRs		54–83% R@20		The approach is based on IR and NLP techniques
	Ren et al. (2019)	Classification task	DupPR (Yu et al., 2018)	83%	11%		The approach is based on IR and NLP techniques
	Zhang et al. (2020)	Recommendation task	The authors constructed a dataset of duplicates (https://github.com/yangzhangs/iLinker)		45–61% R@10		The approach is based on IR and deep learning techniques

The mentioned approaches detect duplicates in developers’ communication channels. To the best of our knowledge, this is the first attempt to related posts in Discussions. Our main challenge is related to the vast diversity of repositories hosted on GitHub, which provide different software project contexts. All these contexts highlight the relevance of designing a method suitable for replication in other GitHub repositories. Therefore, to detect related posts in Discussions forums, our proposed approach (1) does not rely on a pre-labeled dataset; (2) does not rely on domain-specific models; (3) is based on general-purpose deep learning models (a Sentence-BERT pre-trained model), and (4) aims at improving precision rates. In the “The RD-Detector approach” section, we propose a method to detect related posts in Discussions.

## The rd-detector approach

In this section, we present the RD-Detector approach. Portions of this text were previously published as part of a preprint (Lima et al., 2022). The RD-Detector aims to detect pairs of candidates of related posts in collaborative discussion forums of developers. To this end, the RD-Detector scores the Semantic Textual Similarity (STS) between posts to detect duplicate and near-duplicate posts. The greater the similarity values, the greater the chances of duplicates.

We conceptualize duplicate and near-duplicate posts as follows.

- *Duplicate posts* are those with the same content. The duplicate post items (title, description, and author) can be exact or close copies. Posts’ authors can rewrite some items by adding or deleting information. Detecting duplicate posts is essential to mitigate the duplication problem in collaborative discussion forums of developers.
- *Near-duplicate posts* are those posts with similar topics. Different users with similar interests, questions, or ideas create and comment on them. Items of near-duplicate posts (title, description, and author) are not the same but share information related to each other’s content. Detecting near-duplicate posts is essential to disseminate the project knowledge.

We define any duplicate or near-duplicate posts as “related posts.” Throughout the remainder of this article, we will solely use the term “related posts” to refer to them. The RD-Detector provides a set of candidates for related posts, which we will denote as *R* going forward.

**Figure 2:**
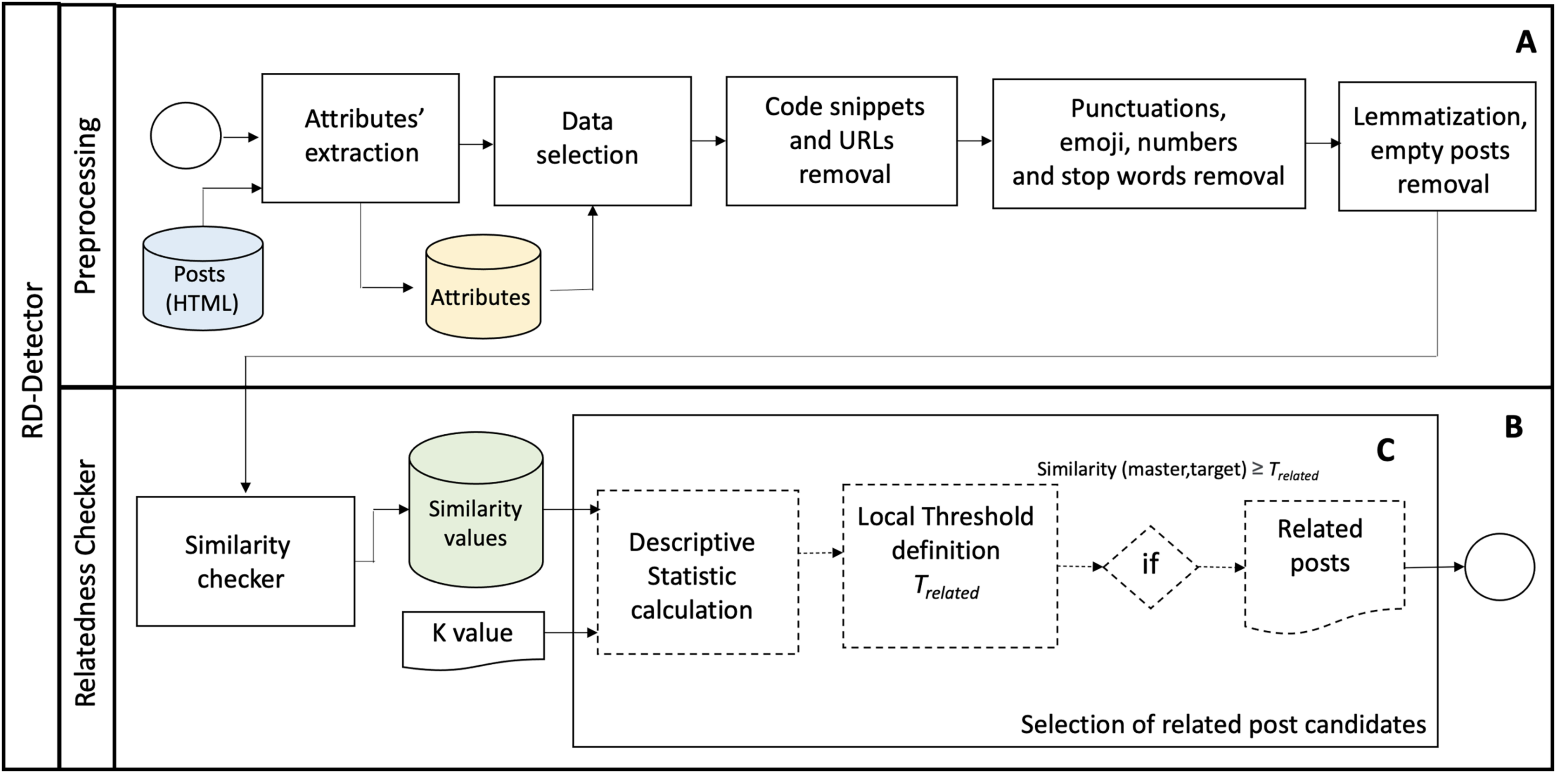
The RD-Detector approach.

Figure 2 shows the overall process conducted to detect the sets of candidates for related posts. The process comprises two phases: Preprocessing (Fig. 2A) and Relatedness Checker (Fig. 2B). We describe the phases of the proposed approach as follows.

### Preprocessing

Applying text similarity algorithms in discussion post contents requires data preprocessing to optimize the algorithm execution (Zhou et al., 2017). In preprocessing, we first extract attributes from each post in the “Attributes’ extraction” step. The RD-Detector uses the attributes’ values to select the posts that should be evaluated. In this research, we use the repository name and the posts’ IDs, categories, authors, dates, and titles as attributes. In the “Data selection” step, the RD-Detector converts the text to lowercase and selects the posts’ title and body text. In addition, the approach filters discussion posts according to the values of their attributes.

The RD-Detector considers the title and the body of the discussion first posts to calculate the semantic text similarity values between pairs of posts. We do not consider comments and replies because they are feedback or design reasoning about the discussion posts’ main topics.

The following two preprocessing steps, “Code snippets and URL removal” and “Punctuation, emoji, numbers, and stopwords removal,” aim to remove noise data from the content of the posts. We remove the code snippets since the machine learning model is a general-purpose model trained to handle NLP problems. Furthermore, the structure and vocabulary of natural language texts differ from machine codes (Sirres et al., 2018). We parsed the HTML document of each post and removed code snippets and URLs embedded in specific HTML tags, *e.g*., <code> and <a href>.

The “Punctuation, emoji, numbers, and stopwords removal” step removes punctuations, emojis, numbers, and stopwords from posts’ content. To this end, we use the Python Natural Language Tool Kit (NLTK) library (https://www.nltk.org/) (Bird, 2006). We do not remove the punctuation symbols ‘.’ and ‘_’ because they concatenate words, *e.g*., ‘Next.js’ and ‘version_1.3’. Finally, we apply lemmatization to the remaining text (Zhang et al., 2017) and eliminate zero-length discussion posts in the “Lemmatization, empty posts removal” step.

### Relatedness checker

The “Relatedness Checker” phase is the core of the RD-Detector (Fig. 2B). In this phase, the approach computes the similarity score of post pairs and detects the candidates for related posts (*R*). The preprocessed data is the input of this phase (Algorithm 1, lines 0–3). *R* is the output of the “Relatedness Checker” phase (Algorithm 1, line 30). This phase comprises two steps: (1) the similarity measurement (“Similarity checker”) and (2) the “Selection of related post candidates.” The steps are as follows.

**Algorithm 1:** Identifying related posts.

**Input:** Set of posts ***D***
** Input:** Set of attributes **Δ**
** Input:** **K** value
** Input:** Sentence-BERT **model**
1**foreach** *discussion* ${d_i} \in$ D **do**
2 $D^\prime \leftarrow D^\prime +\, preprocessing({d_i})$;
3 **end**
4 $$/*{\mkern 1mu} similarity\,Checker*/$$
5 **foreach** *discussion* ${d_i} \in D^\prime {\bf do}$ **do**
6 **foreach** *discussion* ${d_j},({d_j} \ne {d_i}) \in D^\prime$ **do**
7 $maste{r_i} \leftarrow {d_i}$;
8 $targe{t_j} \leftarrow {d_j}$;
9 $similarity\_valu{e_{i\_j}} \leftarrow similarity(maste{r_i},targe{t_j})$ */ ∗ Using **model** ∗ /*;
10 $tuple \leftarrow (maste{r_i},targe{t_j},similarity\_valu{e_{i\_j}})$;
11 $save(tuple,similarity\_values\_file)$;
12 **end**
13 **end**
14 $/*Descriptive\,Statistic\,calculation\,and\,Threshold\,definition*/$
15 $S \leftarrow \{ \}$
16 **foreach** *discussion* ${d_i} \in D^\prime$ **do**
17 $maste{r_i} \leftarrow {d_i}$;
18 $S = S + topK\_sim\_values(maste{r_i},K)$;
19 **end**
20 $Q1 \leftarrow 25th\_percentile(S)$;
21 $Q2 \leftarrow 50th\_percentile(S)$;
22 $Q3 \leftarrow 75th\_percentile(S)$;
23 $IQR \leftarrow Q3 - Q1$;
24 ${T_{related}} = Q3 + (1.5*IQR)$;
25 $/* Selection\,of\,related\,post\,candidates*/$
26 $R \leftarrow \{ \}$;
27 **foreach** $maste{r_i},targe{t_j},similarity\_valu{e_{i\_j}} \in similarity\_values\_file$ **do**
28 **if** $(similarity\_valu{e_{i\_j}} \ge {T_{related}})$ **then** $R \leftarrow R + (maste{r_i},targe{t_j})$;
29 **end**
30 **return** *R*;

#### Similarity checker

We use a semantic text similarity (STS) checker (Agirre et al., 2015) to score the similarity between pairs of posts. The RD-Detector uses the public all-mpnet-base-v2 (https://huggingface.co/sentence-transformers/all-mpnet-base-v2). Sentence-BERT model to compute semantically significance sentence embeddings of each post. The model maps sentences and paragraphs to a 768-dimension dense vector space. The model is designed to encode sentences and short paragraphs. It truncates input text longer than 384 words. At the time of this work execution, the all-mpnet-base-v2 model provided the best quality in sentence embedding computing and semantic searching performance (Reimers, 2021). The all-mpnet-base-v2’s base model is the microsoft/mpnet-base model. Experts optimized the all-mpnet-base-v2 model over 1 billion sentence pairs collected from various datasets (Reddit comments (2015-2018), WikiAnswers Duplicate question pairs, Stack Exchange Duplicate questions (titles+bodies), *etc*.), (Hugging Face, 2021). We used the available version of the all-mpnet-base-v2 model. We did not tune the model to our research context. We used the default parameters.

We compare the sentence embeddings using the cosine similarity score. We used the score to set the similarity value between pairs of posts and to rank sentences with similar meanings. The cosine similarity value outcome is bounded in [0,1]. Cosine similarity value 1 refers to identical posts, and value 0 refers to dissimilar posts. We use ${\rm similarity}\,(master,target)$ to denote the similarity value between a master and a target post. There is no hierarchy or priority relationship between pairs of masters and targets posts. Therefore, the similarity value for ( $maste{r_i}$, $targe{t_j}$) is equal to the similarity value for ( $targe{t_j}$, $maste{r_i}$).

For each pair of a $(master,target)$ posts, the RD-Detector computes the semantic similarity value that captures the relatedness of the posts, Algorithm 1—lines 4–13. The similarity values are the input to the “Selection of related post candidates” step.

#### Selection of related post candidates

We rank the candidates for related post according to their similarity values. The approach uses a threshold value to determine whether two posts are related. However, we do not use a pre-determined threshold $x$ to filter the related posts. Instead, the RD-Detector computes what we call the ‘local threshold,’ denoted by ${T_{related}}$ (Fig. 2C). The ${T_{related}}$ is defined automatically using descriptive statistics that describe the characteristics of a specific similarity value set. Different input datasets have different threshold values. Since the threshold value is based on descriptive statistics, it is possible to reproduce the proposed method in other software contexts. We define the ${T_{related}}$ value following four steps:

1. **Defining the *K* value:** The *K* value delimits the search bounds for related posts candidates. The RD-Detector uses the *K* value to select the similarity values of the top-K most similar posts to each discussion post in the input dataset. The greater the value of *K*, the greater the number of similarity values selected. Setting $K = 3$, the approach uses the similarity values of the top-3 most similar discussion posts to every post in the dataset. Setting $K = 20$, the RD-Detector selects the similarity values of top-20 most similar discussion posts. The *K* value can range from 1 to $n - 1$, where $n$ is the number of discussion posts in the dataset. *K* is an input value (Algorithm 1).
2. **Creating the distribution *S*:** The *S* distribution is a collection of similarity values. *S* contains the similarity values of the *K* most similar target discussion posts for each discussion post in the dataset (Algorithm 1, lines 14–19).
  Let $n$ be the number of discussion posts in the dataset, *K* the number of the most similar target posts to every discussion in the dataset, and $valu{e_{i\_j}}$ the similarity value of a given $maste{r_i}$ and $targe{t_j}$ post pair, the distribution *S* is:
$$\eqalign{  & \quad S = \,\, < valu{e_{1\_1}},valu{e_{1\_2}}, \ldots ,valu{e_{1\_K}},valu{e_{2\_1}},  \cr 
  & \,\,\,\,\,\,\,\,\,\,\,\,\,\,\,\,\,\,valu{e_{2\_2}}, \ldots ,valu{e_{2\_K}}, \ldots ,valu{e_{n\_1}},valu{e_{n\_2}}, \ldots ,valu{e_{n\_K}} >  \cr} $$
3. **Determining the descriptive statistics of *S*:** We use descriptive statistics variability measures to understand how dispersed the distribution *S* is. To this end, we calculate the interquartile range (*IQR*), along with the 25th percentile ( $Q1$), the 50th percentile ( $Q2$), and the 75th percentile ( $Q3$), Algorithm 1—lines 20 to 23. Next, we find the Upper Inner Fence value (Eq. (1)) that identifies the outliers in *S* (Tukey, 1977):
(1) $$Upper\,inner\,fence = Q3 + (1.5*IQR)$$
4. **Setting the local threshold ( ${T_{related}}$):** Because we assume that the greater the semantic similarity value of a pair of posts, the greater the chances they are related posts. We set the local threshold to the upper inner fence value (Algorithm 1—line 24). Therefore, we have that:
(2) $${T_{related}} = Upper\,inner\,fence$$
  The *K* value defines the *S* distribution size. Consequently, it changes the coefficients $Q1$, $Q2$, $Q3$, and *IQR* values that summarize *S*. As a result, it also causes changes in the local threshold value, ${T_{related}}$. Since ${T_{related}}$ is directly influenced by *S*, we call ${T_{related}}$ as ‘local threshold’.

After setting the local threshold, the RD-Detector detects the pairs of candidates for related posts. Related posts are those pairs with similarity values equal to or greater than the local threshold. RD-Detector outputs *R*, the set of related post candidates (Algorithm 1, lines 26-30). We consider related posts those pairs identified as outliers in the *S* distribution. Calefato et al. (2021) also use descriptive statistics to identify core OSS developers’ inactivity periods.

Every time a new post is to be evaluated, its similarity should be evaluated with respect to all previous posts. We evaluated the RD-Detector using the Accuracy, Precision, and Recall metrics (Kim et al., 2005), considering the assessment of three OSS maintainers and three SE researchers who manually classified the sets of related post candidates (*R*). Since we do not know the number of related posts or the truly related posts in the Discussions forums, we created an oracle with posts labeled as related and unrelated to enable us to assess the approach’s recall. We present the overall process of the RD-Detector evaluation in the “RD-Detector evaluation” section.

The RD-Detector emerges as a tool that maintainers can use to minimize the work overhead in manually detecting related posts and reduce the rework in answering the same question multiple times. The periodic execution of RD-Detector supports maintainers in dealing with related post propagation in GitHub communities.

## Assessing rd-detector over github discussions forums

To assess RD-Detector, we collected and used pairs of posts of three selected OSS projects hosted on the GitHub platform (Table 2). RD-Detector creates sets containing candidates of related posts for each project. Maintainers of the selected OSS projects and SE researchers evaluated the RD-Detector outcomes.

**Table 2:** Repositories used in the dataset.

Repository	#Posts
Gatsby (https://github.com/gatsbyjs/gatsby/discussions/)	1,886
Homebrew (https://github.com/homebrew/discussions/discussions/)	2,490
Next.js (https://github.com/vercel/Next.js/discussions/)	11,672
**sum**	**16,048**

### Data collection—GitHub Discussions

We collected all public posts of three OSS communities. We stored the collected data in a local dataset. We downloaded the discussion threads’ HTML files (discussion first post and comments) and compiled a list with the posts’ IDs, categories, authors, dates, and titles (the posts’ attributes).

We collected data from Gatsby ( ${D_{p = Gatsby}}$), Homebrew ( ${D_{p = Homebrew}}$), and Next.js ( ${D_{p = Next.js}}$) repositories (Table 2). We chose these three repositories based on three main criteria: (1) the use of GitHub Discussions, (2) the GitHub Discussions engineering team’s involvement collaboration in contacting maintainers, and (3) the availability of OSS maintainers for evaluation. We collected all public discussion posts created in the repositories’ forums until September 2022. In total, we collected 16,048 posts.

#### Dataset characterization

**Figure 3:**
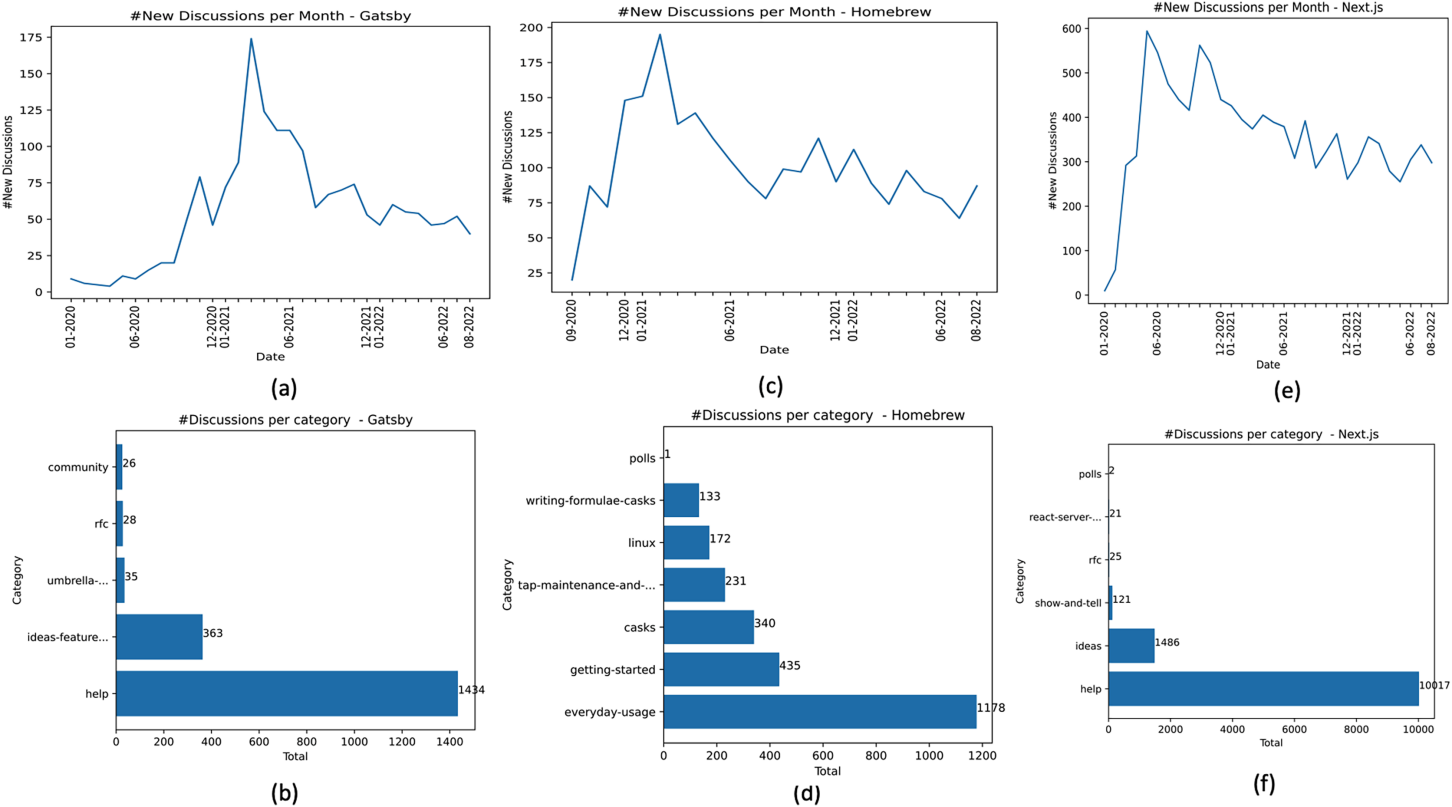
Dataset characterization—Gatsby, Homebrew, and Next.js.

According to the Gatsby community documentation (Gatsby Community, 2022), “Gatsby is a free and open source framework based on React that helps developers build blazing fast websites and apps.” We call ${D_{Gatsby}}$ the set of posts collected from Gatsby’s repository. On the date we performed the data collection, we collected 1,886 posts from Gatsby, $|{D_{p = Gatsby}}| = 1,886$ (Table 2). The Gatsby forum contains repository-level posts, meaning that posts are not visible at the organization level. Figure 3A shows the frequency distribution of posts created in the Gatsby community over 32 months (01-2020 to 08-2022). According to this time window, 01-2020 to 08-2022, the average growth rate of forum usage by the Gatsby community was 13.4% (considering the frequency of new posts). Gatsby makes available the following category types community, help, ideas-feature-requests, RFC, and umbrella-discussions. Help posts are most common, totaling 76.03%, followed by ideas-feature-requests, umbrella-discussions, RFC, and community, totaling 19.24%, 1.85%, 1.48%, and 1.37% of posts, respectively (Fig. 3B).

Homebrew is an OSS project that makes it easy to “install the UNIX tools Apple didn’t include with macOS. It can also install software not packaged for your Linux distribution to your home directory without requiring sudo” (Homebrew Project, 2022). We call ${D_{Homebrew}}$ the set of posts collected from Homebrew’s repository. We collected 2,490 posts from Homebrew, $|{D_{p = Homebrew}}| = 2,490$. Figure 3C shows the frequency distribution of posts created in Homebrew Discussions forum over 24 months (09-2020 to 08-2022). The oldest post collected from Homebrew dates from September 2020. According to data in ${D_{Homebrew}}$, the average growth rate of the forum usage in Homebrew between 09-2020 and 08-2022 was 18.02% (considering the frequency of new posts). Homebrew organizes its discussion threads at the organization level according to the problem types and not by question types. Homebrew provides the following categories: casks, getting-started, tap-maintenance-and-brew-development, everyday-usage, linux, polls, and writing-formulae-casks. The everyday-usage posts are the most common in the ${D_{Homebrew}}$ dataset, totaling 47.30%. Followed by getting-started, casks, tap-maintenance-and-brew-development, linux, writing-formulae-casks, and polls totaling 17.46%, 13.65%, 9.27%, 6.90%, 5.34%, and 0.04% of posts, respectively (Fig. 3D).

Next.js is an OSS project that “provides a solution to build a complete web application with React” (Vercel, 2022). The Next.js community has stood apart in supporting the launch of Discussions since the start (https://github.blog/2022-01-13-how-five-open-source-communities-are-using-github-discussions/). Compared to Gatsby and Homebrew, Next.js is the project with the highest number of posts analyzed, characterizing an active OSS community. The Next.js forum contains repository-level posts. Figure 3E shows the frequency distribution of new posts over 32 months (01-2020 to 8-2022). In this period, the average growth rate of the forum in the Next.js community was 30.43% (considering the number of new posts created). The Next.js project has the highest average growth rate among the three analyzed projects. When we collected the dataset, the discussion categories were ideas, help, polls, react-server-components, RFC, and show-and-tell. Help posts are the most frequent in the Next.js dataset, totaling 85.82% of the posts. Followed by ideas, show-and-tell, RFC, react-server-components, and polls, totaling 12.73%, 1.03%, 0.21%, 0.17%, and 0.01% of posts, respectively (Fig. 3F).

Finally, all posts collected compose the dataset *D* used in this work. Therefore, $D = {D_{p = Gatsby}} \cup {D_{p = Homebrew}} \cup {D_{p = Next.js}}$.

### Preprocessing phase applied to discussions dataset

We preprocessed the dataset *D*. First, we split the dataset into subsets using predefined filters. We considered two filter types: project and category filters. We used the project filter, $p$, to select posts from a specific repository by defining the project’s name. We used the category filter, $c$, to select posts that matched Help and Ideas category types. By setting both filters, we can better evaluate and report the RD-Detector results. Since Help posts tend to have more code snippets than others, it is relevant to consider the posts’ category.

We set both filter values using the discussion post attributes extracted from the input dataset. The category filter values depend on the types of categories provided by the project. We express the use of both filters by $p = \alpha |c = \beta$, where $p$ identifies the project name (ex.: Gatsby, Homebrew, and Next.js) and $c$ identifies the discussion category type.

We standardized the category labels to report our results, making them easier to describe and follow. We use the following labels: Q&A, Ideas, and ALL. The Q&A label refers to ‘Question and Answers’ (help) discussion types. The Ideas label refers to ideas or ideas-feature-requests categories. The Q&A label refers to the help category for both Gatsby and Next.js projects, and the Ideas label refers to the ideas-feature-requests and ideas categories for Gatsby and Next.js projects, respectively. In addition, we created the label “ALL” to refer to all posts from a particular repository, regardless of their original category. For example, the category label ALL comprises all posts pre-classified in ideas, help, polls, react-server-components, RFC, and show-and-tell categories from the Next.js repository. As described in the “Dataset characterization” section, the Homebrew project discussion organization differs from the other projects. Due to this fact, we chose not to filter Homebrew posts according to the category type. Therefore, we report the results of the Homebrew project by setting $c = ALL$.

We considered splitting the dataset into categories because Q&A and Ideas type posts are the majority in both Gatsby and Next.js projects. The union of the Q&A and Ideas types discussion represents 95.28% and 98.55% of the posts in the ${D_{p = Gatsby}}$ and ${D_{p = Next.js}}$ datasets, respectively. In addition, Discussions documentation encourages using both categories for different purposes: Q&A-type posts for asking the community for help on project-specific issues and Ideas for sharing and discussing ideas for new features (GitHub, 2022a).

We derived seven sub-datasets by applying different filter configurations to the datasets (Table 3, column 3). We use ${D_{p = Gatsby|c = Q \,\&\, A}}$ to designate the subset of Q&A posts collected from the Gatsby project, ${D_{p = Gatsby|c = Ideas}}$ to denote the subset of ideas-feature-requests posts collected from the Gatsby project, and so on. We assessed the proposed approach (RD-Detector) over the seven sub-datasets.

**Table 3:** Configuration groups: projects, categories, and K-values.

Project	Category	Dataset	Set of candidates for related posts
Gatsby	Q&A	$D_{P = Gatsby|c = Q\&A}$	${R_{P = Gatsby|c = Q\&A|K = 5}}$ ${R_{P = Gatsby|c = Q\&A|K = 10}}$
	Idea	${D_{p = Gatsby|c = idea}}$	${R_{p = Gatsby|c = idea|K = 5}}$ ${R_{p = Gatsby|c = idea|K = 10}}$
	ALL	${D_{p = Gatsby|c = ALL}}$	${R_{p = Gatsby|c = ALL|K = 5}}$ ${R_{p = Gatsby|c = ALL|K = 10}}$
Homebrew	ALL	${D_{p = Homebrew|c = ALL}}$	${R_{p = Homebrew|c = ALL|K = 5}}$ ${R_{p = Homebrew|c = ALL|K = 10}}$
Next.js	Q&A	$D_{P = Next.js|c = Q\&A}$	$R_{P = Next.js|c = Q\&A|K = 5}$ $R_{P = Next.js|c = QA|K = 10}$
	Idea	${D_{p = Next.js|c = idea}}$	${R_{p = Next.js|c = idea|K = 5}}$ ${R_{p = Next.js|c = idea|K = 10}}$
	ALL	${D_{p = Next.js|c = ALL}}$	${R_{p = Next.js|c = ALL|K = 5}}$ ${R_{p = Next.js|c = ALL|K = 10}}$

Next, the sub-datasets went through cleaning, denoising, and formatting steps. The last preprocessing step ignores zero-length posts. In total, the approach discarded 3, 2, and 6 posts from Gatsby, Homebrew, and Next.js projects, respectively.

### Relatedness checker applied to GitHub Discussions

After the preprocessing phase, the RD-Detector calculated the semantic similarity values for each pair of posts (as presented in the “Similarity checker” section), using the seven sub-datasets presented in Table 3, column 3.

The RD-Detector uses the *K* value to select the similarity values of the top-K most similar posts. In order to evaluate the RD-Detector precision, we set the *K* value to 5 and 10. For each ${D_{p = \alpha |c = \beta }}$ sub-dataset presented in Table 3, we configured the approach to run over $K = 5$ and $K = 10$. This way, we evaluate the approach’s precision by considering the five and the ten most similar posts to every discussion in the dataset. Values greater than 10 expand the search bounds for related posts. We did not consider *K* values greater than ten because the proposed approach aims to increase precision. However, we conducted a second experiment to measure the recall rate of RD-Detector. To do so, we first manually created a labeled sampling of (un)related posts. Then, we evaluated the RD-Detector considering different values of *K*. We expect maintainers to set the value of *K* according to their needs. They can decrease the *K* value to increase the chances of detecting duplicates or increase *K* to examine occurrences of near-duplicates.

In total, we assessed the approach over 14 different configuration groups (Table 3, column 4). For each configuration group, RD-Detector computed the local threshold value, ${T_{related}}$, and detected the set of candidates of related posts, *R*. $R{\rm_{p = Gatsby|c = Q\&A|K = 10}}$ denotes the set of candidates for related posts detected considering the configurations $p = Gatsby|c = QA|K = 10$. One can use the same reasoning for the other 13 sets of related posts in Table 3, column 4. We used the configuration groups to evaluate the RD-Detector and report our results.

### The RD-Detector evaluation

We recruited maintainers of the three OSS projects analyzed (one maintainer per project) and three Software Engineering (SE) researchers to evaluate the RD-Detector outcomes. OSS maintainers and SE researchers manually classified pairs containing candidates of related posts as duplicate, near-duplicate, or unrelated. OSS maintainers judged pairs according to their work project. The SE researchers judged pairs containing candidates of related posts detected for the three projects. The maintainers, M_Gatsby, M_Homebrew, and M_Next.js, were contacted *via* the GitHub engineering team. M_Gatsby actively contributes to Gatsby’s community; he creates content and themes for Gatsby. He has committed to the repository more than 600 times over the past five years. M_Homebrew is Homebrew project leader. He has actively committed to the repository more than 7,000 times over the past 11 years. Finally, M_Next.js is also an active contributor to Next.js’s community; he has committed to the repository more than 500 times. The SE researchers, SE_R1, SE_R2, and SE_R3, have different expertise in software development. SE_R1 is an industry practitioner and a Software Engineering researcher, SE_R2 is an active OSS contributor and a Software Engineering researcher, and SE_R3 is a Software Engineering researcher. All researchers have more than five years of experience in their respective working areas.

Evaluators received online documents containing instructions to evaluate the candidates of related posts detected by the RD-Detector configuring $K = 5$ and $K = 10$. The documents (1) described the concept of duplicated and near-duplicated posts and (2) listed pairs of related post candidates. We characterized each pair of related post by describing the ID and the title of the master and target posts. The ID was a link from which evaluators could access the original posts. We instructed the evaluators to add the label ‘D’ to duplicates, ‘R’ to neaR-duplicated ones, and ‘N’ for uNrelated posts. We also asked the evaluators to add comments to justify their judgments. Although we consider related posts those posts that are duplicated and near-duplicated, we made sure that the evaluators were well-informed about both concepts, as we presented in Section “The RD-Detector approach”.

We measured the RD-Detector precision rate compared to the OSS maintainers’ and SE researchers’ judgment. Maintainers are aware of their entire project. They could judge those pairs of related post candidates that require in-depth project context knowledge. The SE researchers evaluated pairs of well-known related posts of all projects. The researchers also supported evaluating related post candidates for the Next.js project.

## Results

In this section, we present the experimental results of RD-Detector evaluation. We provide evidence to support our research question’s answer.

Tables 4–6 present the local threshold values ( ${T_{related}}$) and the number of candidates of related post (|*R*|) detected for Gatsby, Homebrew, and Next.js projects, respectively. Tables 4–6 also show, for each configuration group (project name, category type, and *K* value), the distribution size ( $size(S)$) and the descriptive statistics coefficients that summarize *S* (*IQR*, $Q1$, $Q2$, and $Q3$). We used the values of the coefficients to calculate the local threshold ${T_{related}}$ (Eq. (2)).

**Table 4:** Descriptive statistics—Gatsby.

	Gatsby—similarity values					
	c = Q&A		c = Ideas		c = ALL	
	$ K = 5$	$ K = 10$	$ K = 5$	$ K = 10$	$ K = 5$	$ K = 10$
*Size(S)*	6,061	12,111	1,425	2,826	7,892	15,729
*IQR*	0.147	0.151	0.134	0.140	0.139	0.142
$Q1$	0.579	0.555	0.513	0.476	0.586	0.560
$Q2$	0.663	0.641	0.593	0.560	0.665	0.642
$Q3$	0.727	0.706	0.648	0.617	0.725	0.703
${T_{related}}$	0.9493	0.9348	0.8498	0.8287	0.9339	0.9181
|*R*|	3	5	6	9	7	9

**Table 5:** Descriptive statistics—Homebrew.

	Homebrew—similarity values	
	c = ALL	
	$ K = 5$	$ K = 10$
*Size(S)*	10,386	20,611
*IQR*	0.128	0.129
$Q1$	0.550	0.525
$Q2$	0.617	0.593
$Q3$	0.678	0.654
* ${T_{related}}$*	0.8717	0.8476
|*R*|	20	34

**Table 6:** Descriptive statistics—Next.js.

	Next.js—similarity values					
	c = Q&A		c = Ideas		c = ALL	
	$ K = 5$	$ K = 10$	$ K = 5$	$ K = 10$	$ K = 5$	$ K = 10$
*Size(S)*	41,426	82,033	5,832	11,476	48,120	95,327
*IQR*	0.110	0.113	0.112	0.112	0.108	0.110
$Q1$	0.589	0.567	0.560	0.532	0.597	0.574
$Q2$	0.647	0.625	0.619	0.590	0.653	0.632
$Q3$	0.700	0.680	0.673	0.644	0.705	0.685
* ${T_{related}}$*	0.8650	0.8501	0.8415	0.8130	0.8679	0.8509
|*R*|	132	175	90	151	220	309

We can note that as we increase the value of *K*, the local threshold values, ${T_{related}}$, decrease (Tables 4–6). Since the local threshold value decreases, RD-Detector detects new pairs of related posts. The new pairs are those outliers considered by changing the Upper Inner Fence value (Eqs. (1) and (2)).

Table 4 presents the results of the Gatsby project. Considering $K = 5$ and $c = \rm Q\& A$, RD-Detector calculated the local threshold value based on the similarity values of 6,061 unique pairs of posts, $size(S)$, setting ${T_{related}} = 0.9493$ and detecting three pairs of related post candidates, |*R*|. Comparing the local threshold ${T_{related}}$ between Q&A and Ideas, we can notice the Ideas local threshold are smaller for both $K = 5$ and $K = 10$, suggesting the need to investigate the difference between Q&A and Ideas posts.

Table 5 presents the RD-Detector outcomes for the Homebrew project. The RD-Detector almost duplicated the number of posts evaluated to detect 14 new candidates of related posts when we set the approach to run using $K = 5$ and $K = 10$. Additionally, the threshold value decreased by 0.024 units. Although the number of evaluated pairs was duplicated, the number of candidates for related posts was not. This suggests that increasing *K* also increased the number of unrelated post instances in the distribution *S*.

Table 6 presents the distribution size, $Size(S)$, and the number of detected related posts, |*R*|, for the Next.js project. These numbers endorse the scenario described by Ahasanuzzaman et al. (2016). The authors highlighted that the number of posts increases as forums become popular. Consequently, it may also increase the number of duplicates and near-duplicate posts. Since the Next.js project is the largest considering the number of posts (Table 2), the chances of related posts occurring can increase. Using the configuration group $|p = Next.js|c = ALL|K = 10$, the RD-Detector detected 309 pairs of candidates for related posts—the most extensive set detected (Table 6).

**Figure 4:**
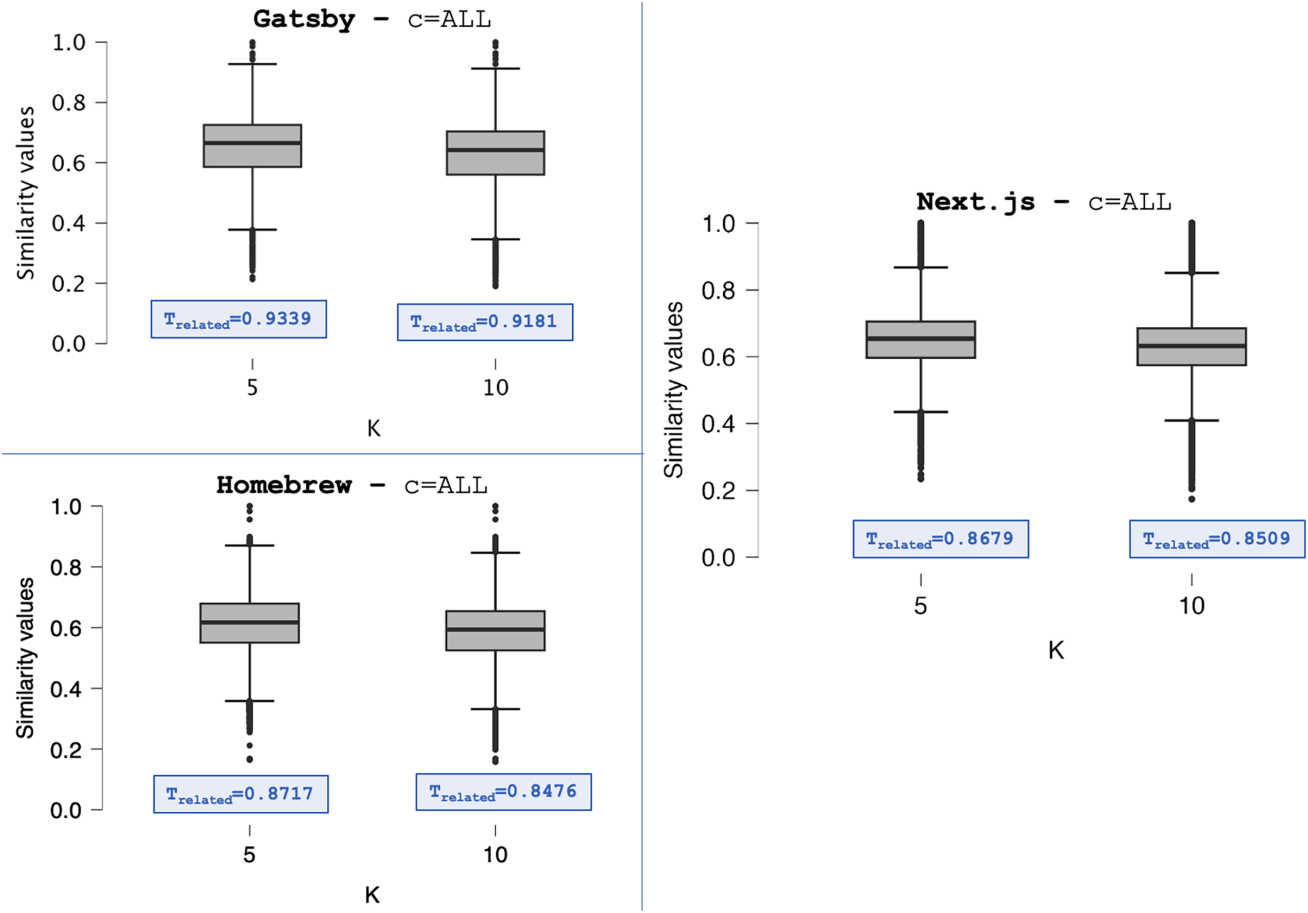
The *S* distributions (*C* = *ALL*)—Gatsby, Homebrew, and Next.js.

Figure 4 displays the Boxplots of the distributions *S* from Gatsby, Homebrew, and Next.js repositories, setting the category filter to $c = ALL$ and the *K* value to $5$ and $10$. The distributions shown in Fig. 4 are summarized in Tables 4–6. Despite being discrete, the median values show changes in the centrality tendencies of *S* distributions. When setting $K = 10$, the centrality tendencies of the distributions are lower compared to $K = 5$. This difference indicates the addition of post pairs with lower similarity values in *S*. We observed this pattern in all distributions summarized in Tables 4–6.

For each of the 14 configuration groups presented in Table 3 column 4, the RD-Detector calculated different values of local thresholds and detected different amounts of candidates of related posts. We highlight that all sets of candidates for related posts detected considering $K = 5$ are subsets of the group of related posts considering $K = 10$. Table 7 shows the precision rate reached by the RD-Detector considering the 14 configuration groups, considering the thresholds described in Tables 4–6. We highlight that RD-Detector is a parametrizable approach; maintainers can set the *K* value according to their interest in detecting duplicates or near-duplicates.

**Table 7:** Evaluation results—precision.

Category	*K* = 5	*K* = 10
	Gatsby	
Q&A	100% (3/3)	100% (5/5)
Ideas	83.33% (5/6)	77.78% (7/9)
ALL	100% (7/7)	100% (9/9)
	Homebrew	
ALL	95% (19/20)	91.17% (31/34)
	Next.js	
Q&A	93.94% (124/132)	89.14% (156/175)
Ideas	98.89% (89/90)	95.36% (144/151)
ALL	99.09% (218/220)	93.85% (290/309)

The OSS maintainer, M_Gatsby, and two SE researchers (SE_R1 and SE_R3) evaluated the related post candidates for the Gatsby project. In order to save M_Gatsby’s time and effort in judging cases of duplicates that are exact copies, the GitHub engineering team requested researchers to judge pairs of related posts with a high similarity value. Those pairs are well-known duplicates because they are exact copies. The two SE researchers judged candidates of related posts with similarity values greater than or equal to 0.9415. In total, SE researchers judged five candidates for related posts. The researchers agreed that all judged pairs contained related posts. The maintainer M_Gatsby judged 13 candidate pairs that required prior technical knowledge about the project. According to Table 7, RD-Detector reached the maximum precision value (100%) in detecting related post pairs fixing $c = Q\&A$. The Ideas category of the Gatsby project had lower precision than other categories. Analyzing Ideas category false-positive predictions, we noticed that the RD-Detector did not capture the project-related issue specifics of two pairs. Although the two candidates of related posts addressed the same topic and had an intersection of project keywords, they addressed different problems.

The maintainer M_Homebrew and two SE researchers (SE_R1 and SE_R3) evaluated the related post candidates for the Homebrew project. The researchers judged pairs of related post candidates with similarity values greater than or equal to 0.9558. In total, SE researchers evaluated four pairs of well-known duplicates. The researchers agreed that all four pairs contained related posts. The maintainer M_Homebrew judged 30 candidate pairs. Evaluators judged 19 (out of 20) candidates for related posts presented in ${R_{p\,=\,Homebrew|c\,=\,ALL|K\,=\,5}}$ as related. From the evaluators’ perspective, 31 out of 34 pairs are true positive related posts. The maintainer M_Homebrew judged the posts of three pairs as unrelated. The Homebrew does not provide the same default post categories provided by Gatsby and Next.js. That is why we did not split the Homebrew dataset by category and present the achieved precision rate setting the category filter to *ALL* ( $c = ALL$).

Finally, two SE researchers (SE_R2 and SE_R3) evaluated the related post candidates for the Next.js project. To ensure the reliability of the reported results, we measured the researchers’ inter-rater agreement using the Cohens Kappa Coefficient (Cohen, 1960), which was 0.85. This value indicates almost perfect agreement according to the interpretation proposed by Landis & Koch (1977). However, we collected the Next.js maintainer (M_Next.js) feedback regarding a random sample of 27 pairs of (un)related posts.

Table 7 also shows that precision rates degrade as we increase the value *K*. The RD-Detector identified 18, 34, and 344 different pairs of related post candidates for the Gatsby, Homebrew, and Next.js projects, respectively. Regarding Gatsby project results, two pairs of related post candidates were classified as unrelated by M_Gatsby maintainer. Thus, 16 out of 18 detected pairs were indeed related, achieving 88.88% of accuracy. From the perspective of the maintainer M_Homebrew, out of the 34 related post candidates detected, three are false positives. Therefore, RD-Detector accurately predicted 91.17% truly related posts. Finally, RD-Detector accurately predicted 91.86% of related posts considering the Next.js project. Out of 344 detected pairs, 28 are false positives. We discuss the false positive predictions in the “Discussions” section.

We also measured the recall rate of RD-Detector by calculating the ratio between the true positives returned by the approach and the ground truth positives identified for each project. Ground truth positives are the sum of true positives and false negatives predictions (Kim et al., 2005).

To do this, we selected, for each project, a sample of approximately 400 post pairs whose similarity was calculated by the RD-Detector, considering all posts in the projects’ databases ( $c = ALL$) and setting $K = 10$. We randomly selected the post pairs.

We determined the sample size by calculating a representative sample of posts in each repository. We considered an error margin of 5% and a confidence level of 95%. As a result, we achieved sample sizes of 320, 333, and 372 for Gatsby, Homebrew, and Next.js, respectively. However, we decided to round up each sample size to approximately 400 (409, 418, and 432 for Gatsby, Homebrew, and Next.js) and have made them available in the reproduction package (Lima, 2023). In total, we selected nearly 1,200 pairs of posts. Two SE researchers independently conducted manual classification for each pair in our sample, thereby establishing the ground truth sample for each project. We quantified the agreement between the researchers involved in the manual classification to minimize bias and classification errors using Cohen’s Kappa measure Cohen (1960). The Cohen’s Kappa values achieved were 0.90 for Gatsby, 0.62 for Homebrew, and 0.85 for Next.js project. The values show agreements between the evaluators, which are almost perfect, substantial, and almost perfect (Landis & Koch, 1977). The ground truth positives are 34 for Gatsby, 60 for Homebrew, and 104 for Next.js. The fractions of related pairs within their respective universes represented by the samples are low. For the Gatsby project, only 8.3% of the pairs in the sample are related, 34 out of 409 pairs. For the Homebrew project, 14.35% of the pairs in the project sample are related, which amounts 60 out of 418 pairs. Moreover, only 24.07% of pairs from the Next.js sample are related, amounting 104 out of 432 pairs.

Table 8 presents the achieved recall rates. The three projects exhibited similar behavior concerning Precision and Recall metrics based on the analyzed samples. As the recall increases, the precision decreases. We confirmed that the variation in this behavior is influenced by the value of *K*. We noticed that recall rates greater than 60% are reached when setting $K = 120$ for Gatsby and $K = 40$ for the Homebrew project (Table 8). The *K* value allowed a precision rate greater than 60% in both projects. These recall values denote better-than-random retrieval but less-than-perfect recall performance (Buckland & Gey, 1994). However, the Next.js project did not achieve a 60% in recall value, meaning that the RD-Detector did not detect all true positive related posts. We made available the labeled sample (un)related pairs of discussion posts in the reproduction package (Lima, 2023).

**Table 8:** Evaluation results—sample analysis.

*K*	|*R*|	$ Precision$	$ Recall$
	Gatsby		
5	3	100% (3/3)	8.82% (3/34)
10	4	100% (4/4)	11.76% (4/34)
20	5	100% (5/5)	14.70% (5/34)
30	6	100% (6/6)	17.64% (6/34)
40	7	100% (7/7)	20.58% (7/34)
50	9	100% (9/9)	26.47% (9/34)
60	10	100% (10/10)	29.41% (10/34)
70	11	100% (11/11)	32.35% (11/34)
80	14	100% (14/14)	41.17% (14/34)
90	16	100% (16/16)	47.05% (16/34)
100	19	89.47% (17/19)	50.00% (17/34)
110	21	85.71% (18/21)	52.94% (18/34)
120	29	72.41% (21/29)	61.76% (21/34)
	Homebrew		
5	4	100% (4/4)	6.66% (4/60)
10	6	100% (6/6)	10.00% (6/60)
20	23	95.65% (22/23)	36.66% (22/60)
30	42	78.57% (33/42)	55.00% (33/60)
40	60	66.66% (40/60)	66.66% (40/60)
	Next.js		
5	16	100% (16/16)	15.38% (16/104)
10	24	100% (24/24)	23.07% (24/104)
20	42	76.19% (32/42)	30.76% (32/104)
30	60	65.00% (39/60)	37.50% (39/104)
40	75	60.00% (45/75)	43.26% (45/104)

Finally, we measured the time to create the sentence embeddings of all posts and the time to calculate all the similarity rates between all posts. Table 9 shows the RD-Detector run time considering $K = 10$ and $c = ALL$. The execution time depends on the number of posts. Since the Next.js project is the biggest one, the approach spent much more time on Next.js analysis than the others. We report the time measure in seconds(s). We used a 2,3 GHz Intel Core i5 Dual-Core with 8 GB machine to compute these values. However, we argue that it is unnecessary to recreate all sentence embedding every time the RD-Detector runs. One can save the sentence embeddings for further use. To detect the presence of related posts in the community forum, maintainers should run the RD-Detector periodically, ideally once or twice a week.

**Table 9:** RD-Detector time execution— $ c = ALL$ and $ K = 10$.

#Discussion posts	Embedding creation (s)	Similarity calculation (s)
Gatsby		
1,883	645.55	488.55
Homebrew		
2,488	495.51	557.12
Next.js		
11,666	1,833.03	1,854.15

**Answering our RQ:** Are general-purpose deep machine learning models applied to Natural Language Processing (NLP) problems effective in detecting related posts in the GitHub Discussions?

Approaches based on general-purpose Sentence-BERT models applied to Natural Language Processing (NLP) problems can effectively detect related posts in GitHub Discussions forum. The results presented in Tables 7 and 8 demonstrate the precision and recall rates achieved by employing the all-mpnet-base-v2 model to compute the sentence embeddings of the discussion posts and detecting related posts (including duplicates and near-duplicates) using the cosine-similarity score.

## Discussions

In this section, we discuss the effects of changing the *K* value and the false-positives predictions. Next, we describe the maintainers’ feedback about the RD-Detector practical applications. Then, we compare RD-Detector’s relative measurements with research described in the “Related work” section. Finally, we discuss the implications of this research.

### The impacts of changing the *K* value

By configuring the RD-Detector to run over $K = 5$ and $K = 10$, we note that the sets containing candidates of related posts created when we set $K = 5$ are subsets of $K = 10$. Therefore, there is a risk of false-positive predictions propagation. We identified this propagation problem by analyzing the sets containing candidates of related posts created using the configuration groups $p = Gatsby$ and $c = Ideas$. The same unrelated post pair $(master,target)$ occurs in ${R_{p = Gatsby|c = Ideas|K = 5}}$ and ${R_{p = Gatsby|c = Ideas|K = 10}}$. Although the precision rate tends to decrease, the approach detects new pairs of related posts when we vary the *K* values from 5 to 10.

Lower *K* values restrict the search space and increase the local threshold value, resulting in higher precision but compromising the detection of all related post pairs. Low *K* values are beneficial for identifying duplicates, providing evidence for maintainers to remove posts from the community forum. In this scenario, high recall may not be necessary, as maintainers typically seek only related posts that might be duplicates. Conversely, higher *K* values expand the search space for related posts and reduce the local threshold value, detecting new pairs of related posts and compromising the approach precision. Maintainers can set high *K* values to evaluate the posts comprehensively. In this case, maintainers might use the RD-Detector results to iterate through the posts (Buckland & Gey, 1994) to detect topics commonly discussed and plan proper interventions. Maintainers interested in conducting a deep analysis of the posts can vary the *K* value according to their needs. A deep discussion regarding the impacts of changing the *K* value can be found in the reproduction package (Lima, 2023).

### False-positive RD-Detector predictions

Four authors of this article manually analyzed the false positives. We identified some limitations of the proposed approach. We describe the reasons for the false positive predictions based on evidence extracted from the posts.

We analyzed the false-positive predictions and noted that the approach identified posts with similar topics. However, it failed to capture the project issue specificity. We observed that the RD-Detector could fail to treat particular contexts of software projects, suggesting that related posts may address the same project feature but differ on the issue’s specificity. We will call this limitation the “project-specific limitation.”

We also noticed that the posts’ creators used screenshots to detail or describe the issues and used error log descriptions to show the stack trace of where the error occurred. In addition, we observed the predominance of templates’ and projects’ keywords in the false-positive predictions. The RD-Detector removes screenshots and error descriptions embedded in HTML tags during preprocessing. The approach does not use images or error log descriptions as a source of evidence. However, removing both the screenshots and log errors may eliminate the problem specificity. In addition, after preprocessing, the project’s keywords may stand out against the actual post content. We identified the predominance of the template keywords in the false-positive pairs.

Based on this analysis, we can propose improvements to the proposed approach. For example, we can use the maintainers’ judgments to optimize the classifier by providing related and non-related post samples. Furthermore, we can design strategies to minimize project-specific limitations by treating the predominance of project and template keywords and considering screenshots and log error descriptions as sources of evidence.

### RD-Detector practical applications

We asked maintainers to comment on their decision regarding the candidates for related posts (duplicates and near-duplicates). Based on their comments, we could identify practical applications of RD-Detector.

1. to combine the posts’ content merging the related posts—“These (post) could have been combined into one discussion and it would have made sense…” (M_Homebrew), “…if it were up to me, they should have gone together in the same discussion.” (M_Homebrew).
2. to move a post content to another location reorganizing the discussion threads as comments to each other—“…the new issue should probably have been posted as a comment in the master discussion” (M_Homebrew), “…(the posts) would’ve received better traction as a comment on one another.” (M_Next.js), “This discussion could’ve sufficed as a comment on 21633.” (M_Next.js).
3. to recruit collaborators for specific tasks—“…could be useful though for people looking for other guides to contribute to (in this instance).” (M_Gatsby).

Although maintainers reported some practical applications for RD-Detector, the approach does not aim to merge or organize posts automatically. However, we envision that the RD-Detector can support maintainers in the decision-making process of merging or organizing the posts by detecting related ones.

The variety of projects on the GitHub platform provides opportunities to develop innovative approaches to detect candidates of related posts in Discussions. However, the significant number of communities and the projects’ singularity challenge developing and validating such systems. Those reasons endorse the RD-Detector design decision to use a general-purpose machine learning model and calculate local threshold values.

### Contrasting the RD-Detector measurements

In this section, we compare the relative accuracy, precision, and recall measures obtained by RD-Detector with the methods presented in the “Related work” section. We compare the measurement rates reported in the cited articles with the achieved rates by RD-Detector. We do not focus on identifying which approach is better. We intend to provide evidence of how RD-Detector fits to detect duplicate and near-duplicate posts using a Sentence-BERT pre-trained general-purpose model.

Note that some research evaluated the proposed approaches using precision@k and recall@k measures. Both metrics are typically used to measure the relevance of the top $k$ results to the user’s preferences or needs, especially in scenarios where the system is expected to provide a ranked list of items or results (ranking problems). It should be pointed out that the value of *K* in this study has a different interpretation than previously explained $k$ (precision@k and recall@k). We use the *K* value to delimit the search bounds for related posts candidates and create the *S* distribution. The RD-Detector uses the *K* value to select the similarity values of the top-K most similar posts to each discussion post in the input dataset. The greater the value of *K*, the greater the number of similarity values selected. RD-Detector was not evaluated based on the *K* top recommendations. We aim to assess the entire set of related candidates returned by the approach, as we do not consider *R* to be a ranked list of related posts.

First, we contrasted RD-Detector accuracy with the accuracy values reported by research that aims to identify duplicates in issue and bug-tracking systems. The RD-Detector accuracy varies between 88% (Gatsby) to almost 92% (Homebrew and Next.js). Alipour, Hindle & Stroulia (2013), Kukkar et al. (2020), and Cooper et al. (2021) reported that their approach achieved 88%, 85–99%, and 84% of accuracy, respectively. The range of values achieved suggests the usefulness of the RD-Detector in identifying duplicates. In addition, Cooper et al. (2021) claim that humans can save 65% of the time spent manually detecting duplicates using their approach. Although we do not have this measure regarding the use of RD-Detector, we believe our approach can also reduce the time spent by maintainers in identifying related posts.

Second, we contrasted the precision and recall relative values achieved by RD-Detector with approaches that identify duplicates in developers’ Q&A forums. Zhang et al. (2015)’s proposed approach, DupPredictor, achieved a 63.8% recall rate. Mizobuchi & Takayama (2017)’s approach achieved recall values ranging from 15.48 to 43.13%. Zhang et al. (2018)’s approach achieved recall between 66 and 86%. Wang, Zhang & Jiang (2020) achieved the best values for recall@20, ranging from 76 to 79%. Finally, Pei et al. (2021) reported achieving a recall of 82.28%. We can notice that the strategies vary regarding the reported recall. According to Table 8, the RD-Detector achieved a recall rate of almost 67% for the Homebrew project. We believe this value is because the RD-Detector uses a general-purpose model. All research contrasted uses pre-labeled bases to train or optimize machine/deep learning models. Creating a labeled dataset for Discussions is a research opportunity. However, the dynamism and variety of software contexts on GitHub are challenging to create such a dataset. The labeled dataset we created to measure the RD-Detector recall rate is available in the reproduction package (Lima, 2023).

Finally, we contrasted the relative recall values of approaches that aim to deduplicate issues and pull requests within the GitHub platform with the RD-Detector achieved recall rates. We observe that the RD-Detector can support maintainers in detecting duplicate or near-duplicate posts in Discussions. Yu et al. (2018) reported achieving 70% recall, Li et al. (2017)’s approach achieved between 55.3 and 71%, and Zhang et al. (2020) reported achieving between 38–51% recall@5 and 45–65% recall@10.

Although the recall rate is a relevant metric, we focus on developing a more conservative approach that detects true positive related posts to support the decision-making process of maintainers. Besides, the RD-Detector does not make automatic interventions such as deleting and merging posts. In addition, a conservative approach prevents maintainers from spending a long time moderating truly related posts. Given that RD-Detector is a parameterizable approach, maintainers can adjust the value of *K* (increase or decrease) to expand or compress the search space for related posts and, consequently, change the threshold value used to detect related post pairs. In this case, maintainers can configure the RD-Detector according to their respective time availability and interest to analyze pairs of related post candidates, choosing between a more conservative (better precision) or a more exploratory approach.

Based on the precision values reported in Table 7 and the achieved recall rates in Table 8, we claim that RD-Detector is suitable for detecting related posts (duplicate and near-duplicate posts) in Discussions. However, we acknowledge that the recall reported in Table 8 is low for $K = 5$ and $K = 10$. Our objective is to enhance the approach’s precision and recall values. To achieve this, we intend to upgrade RD-Detector by incorporating code snippets and screenshots that users share in posts during the similarity measurement of posts.

### The implications of this research

This research enables opportunities for maintainers and software engineering researchers as follows.

*Maintainers*: previous research shows that maintainers need to manage multiple aspects of the projects to ensure that the projects’ vision endures (Guizani et al., 2022) and the projects’ long-term sustainability (Dias et al., 2021). To do so, maintainers perform different activities encompassing code and non-coding tasks (Dias et al., 2021; Trinkenreich et al., 2021). Such responsibilities intensify the workload of maintainers. Tan & Zhou (2020) highlight proposing tools as a best practice to decentralize maintainers’ responsibilities. In addition, Dias et al. (2021) report that “to sustain a long-term vision of the project, maintainers should delegate tasks.” In this way, the RD-Detector emerges as an alternative tool to alleviate the maintainers’ labor-intensive task of detecting related posts.

*Research opportunities*: to address the diversity and uniqueness of communities hosted on GitHub, the RD-Detector is based on a Sentence-BERT general-purpose machine learning model. Besides, the approach uses descriptive statistics to calculate the local threshold to detect related posts. Given that software projects are unique development ecosystems, we can not use a single and universal threshold to detect related posts. Research opportunities arise to understand how communities use Discussions and how post categories differ.

## Limitations

Although we proposed a parameterizable approach based on general-purpose machine learning models and descriptive statistics, this research may likely present limitations.

We are aware of the diversity and uniqueness of the communities hosted on GitHub. Since we assessed RD-Detector over three OSS communities, more experiments are needed to assess the results in different communities. However, the GitHub engineering team (coauthors in this research) singled out the selected communities to minimize this limitation. In addition, the dataset refers to a specific time window that does not reflect the current moment of GitHub Discussions forums. However, the Next.js project stands out as it has a high use rate of the forum.

Moreover, judging the relatedness between posts is subjective and could introduce biases in evaluating the RD-Detector. The RD-Detector evaluation presents some challenges: (1) people who judge the relatedness of the posts need to analyze the content of the posts semantically; (2) judging the technical aspects of the posts requires prior knowledge of the project; and (3) the judgment involves human (in)precision regarding the concept of relatedness, although we defined the concept of related posts, the interpretation depends on the evaluators’ perspective. To minimize this threat, we introduced the “related posts” meaning to OSS maintainers and SE researchers before classifying related post candidates. We also contacted selected OSS maintainers to judge the candidates for related posts.

The local threshold calculation can also be a limitation. We consider related posts those pairs identified as outliers in a distribution *S*. *S* contains the similarity values of the *K* most similar target posts for each post in the dataset. As we increase the value of *K*, the median of the distribution decreases, and so does the local threshold value. However, we focused on improving the RD-Detector precision. Higher precision values ensure greater assertiveness in detecting related posts. So, we set the *K* value to 5 and 10. The definition of the *K* value is also a limitation. The RD-Detector maximizes the precision rate by setting the *K* value near 1. As the value of *K* increases, the search space for similar posts gets more extensive, and the approach may detect new pairs of related post candidates, increasing the recall rate; however, the RD-Detector precision rate degrades. We contrasted relative values of RD-Detector evaluation with relative values of other approaches evaluation. Since, to the best of our knowledge, this is the first attempt to detect related posts in Discussions, we are not focusing on deciding which approach performed better.

In addition, we measured the RD-Detector’s recall rate based on a sample of 400 pairs of candidates of related posts selected for each repository. We are aware of the vast number of projects hosted on GitHub, the vast number of different software contexts, and the challenges of creating labeled training datasets. Although the recall rates achieved 66%, the achieved precision values (Table 7) show that RD-Detector is effective in detecting related posts in Discussions. We envision maintainers setting the approach’s parameters according to their needs.

Finally, the RD-Detector considers only the discussion’ first posts to measure the similarity between pairs of posts. We did not include the discussion threads in the comparison (comments and replies). We argue that comments and replies are feedback or design reasoning about the posts’ main topics. However, further analysis should be done. Besides, the model truncates input text longer than 384-word pieces. We analyzed the length of the texts in the datasets to identify the percentage of posts that do not meet the criterion of 384 words or less. We found that 21, 15, and 38 posts in the Gatsby, Homebrew, and Next.js datasets have more than 384 words, respectively. These values represent less than 0.70% of each project’s total posts. Although it does not represent a problem for our study, it could be in forums with longer posts.

## Conclusion

In this work, we presented the RD-Detector, an approach to detect related posts on GitHub Discussions. We assessed RD-Detector over public discussions collected from three OSS communities. In total, the approach evaluated the semantic similarity of 16,048 posts. Three OSS maintainers and three SE researchers judged the detected related posts. We measured the RD-Detector precision rate using OSS maintainers’ and SE researchers’ judgment. Our results show that we can use a general-purpose deep machine learning model applied to NLP problems to detect related posts in Discussions. The RD-Detector achieved an average precision rate of 92.47%, considering the top-10 most similar discussion pairs for each discussion post in dataset *D* ( $K = 10$). Based on a labeled sample, we evaluated the recall rate of the RD-Detector, which reached approximately 67% for the Homebrew project.

The RD-Detector uses a Sentence-BERT (SBERT) pre-trained general-purpose model to compute semantically significant sentence embeddings of posts and the cosine similarity to compare pairs of posts. Publicly available machine learning models bring flexibility to the approach. As researchers release new better models, one can update the RD-Detector. In addition, general-purpose models usage provides advantages such as (1) no need for local complex computational structures for training, validation, and testing of models, (2) no need for model parametrization, and (3) no need for model retraining, revalidation, and retest whenever the context change (Polyzotis et al., 2017; Zhou et al., 2017; Schelter et al., 2015; Lee & Shin, 2020). The dynamism with which communities grow justifies general-purpose machine learning model usage.

The RD-Detector calculates local threshold values to detect candidates of related posts. We use descriptive statistics to calculate the upper inner fence value of a distribution containing the similarity values data of the *K* most similar target discussion posts to each post under processing. We consider related posts those pairs in which the similarity values are outliers in the distribution. This design decision makes the RD-Detector applicable in different software contexts.

The approach yielded different outputs, as shown in Tables 4–6. The numbers of detected related posts highlight the need to investigate how communities use Discussions forum.

Maintainers can benefit from RD-Detector to address the labor-intensive task of manually detecting related posts. In addition, maintainers can benefit from the approach to prioritize the development or update project-specific issues frequently discussed, control the propagation of related posts, and support project knowledge sharing. According to the OSS maintainers’ feedback, one can merge related posts or convert related posts as comments on one another.

We reported and discussed our results with the GitHub engineering team. Our findings showed a real need to plan and tackle related posts on GitHub Discussions. Consequently, the GitHub engineering team is testing some changes to the Discussions interface. In addition to providing the discussion title, body text, and category, users must confirm they have searched for similar threads before creating new posts.

As the next step, we intend to implement RD-Detector as a Bot to run over the GitHub Discussions data to report related post occurrences. We also envision assessing the RD-Detector efficacy in different forums. In addition, we believe this research also brings opportunities to enable project knowledge acquisition and transfer by providing users with project-related issues and making the projects’ knowledge easy to find. Finally, our results can enable project knowledge reuse as users can access related posts that have already been asked and answered, and the project knowledge categorization by identifying similar documents.

## Additional Information and Declarations

### Data Availability

The following information was supplied regarding data availability: The data is available at GitHub and Zenodo: - https://github.com/marcia-lima/RD-Detector/tree/v.1-beta. - marcia-lima. (2023). marcia-lima/RD-Detector: v.1 (v.1-beta). Zenodo. https://doi.org/10.5281/zenodo.7982576.
